# Bilateral Multicentric Paraganglioma

**DOI:** 10.1016/S1808-8694(15)30093-8

**Published:** 2015-10-19

**Authors:** Adriano Sérgio Freire Meira, Lauro Otacílio Campos de Sousa, Camila Gomes Fernandes de Sousa, Ricardo Marcio Morais, Pedro de Oliveira Cavalcanti Filho, Jose Walter da Fônseca Júnior, Jose Diniz Júnior

**Affiliations:** a Resident Physician at the Pedro Cavalcanti Clinic/ UFRN University Hospital; b Resident Physician at the Pedro Cavalcanti Clinic/ UFRN University Hospital; c Medical Student - UFRN; d Resident Physician at the Pedro Cavalcanti Clinic/ UFRN University Hospital; e MD. ENT. Head of the Medical Residency Service at the Pedro Cavalcanti Clinic/ UFRN University Hospital; f MD. ENT at the Pedro Cavalcanti Clinic/ UFRN University Hospital; g PhD in Otorhinolaryngology, Adjunct Professor of Otorhinolaryngology - UFRN. Clínica Pedro Cavalcanti / Hospital Universitário da UFRN

**Keywords:** bilateral, neck, paraganglioma, temporal bone

## INTRODUCTION

Paragangliomas are tumors that originate from microscopic structures associated with the Central Nervous System (CNS) in the para-axial regions of the trunk, neighboring the branchiometric arches and CNS periphery, the paraganglia^1^.

## CASE REPORT

N.F.S., 76, black, complained of dizziness that did not improve with medication, pulsatile tinnitus and progressive hearing loss for the past 3 years in his right ear, as well as left side neck bulging for 30 years, of slow growth, painless, of fibroelastic material, non-pulsatile.

On CT scan we noticed an extensive solid mass with ill-defined lobulated contours, occupying the carotid space in both sides, with right side temporal bone destruction by a tumor radiologically equal to the one previously described, and the patient was allergic to iodine. MRI showed hypervascularized lesions (see Figure), in the aforementioned regions, thus corroborating CT findings. Angiography confirmed the diagnosis.

## DISCUSSION

Neck paragangliomas, because of their rare occurrence, are a difficult to diagnose and treat pathology. Among other causes of neck bulging we have: branchyal cysts, carotid aneurisms, neurofibromas, parotid tumors, tumor metastasis, inflammatory lymphadenopathy, amongst others.

They are mostly benign; paragangliomas can have malignant transformation in 2 to 10% of cases, accounting for 0.012% of head and neck tumors. The most frequent site is the carotid body; the less common sites are the middle ear, jugular, vagus nerve, larynx and aortic arch^2^.

**Figure:**
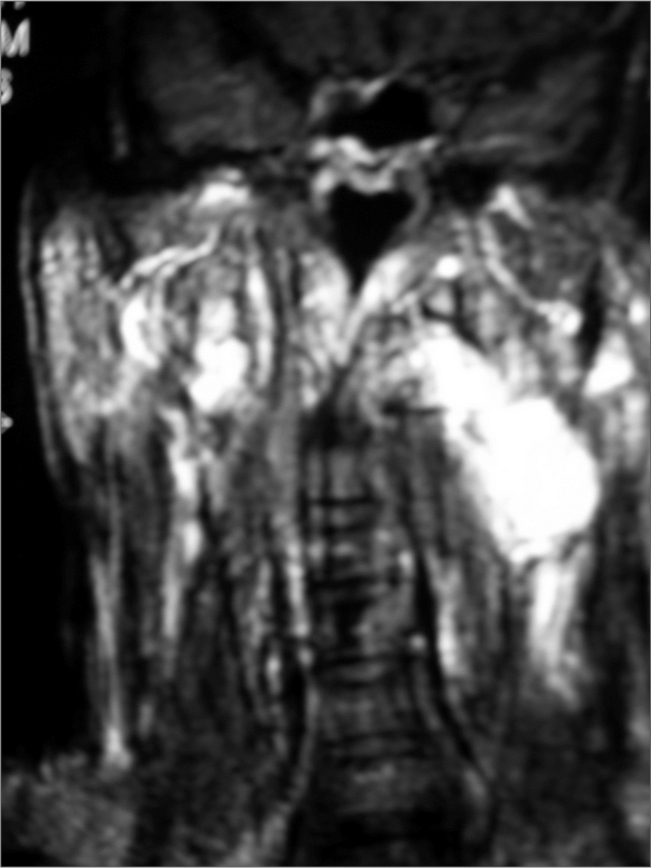
**MRI-** Bilateral neck and jugular-tympanic tumors, more pronounced on the left side.

CT scan is the most adequate diagnostic method for obtaining data on the tumor’s extension and location. MRI is considered superior to CT in documenting soft tissue details and tumor characterization^3^. Nonetheless, only selective angiography provides the definitive diagnosis, outlines the structure and looks for synchronic tumors. FNA should not be done; these tumors rarely secrete catecholamine, however one can look for vanyl-mandelic acid and urinary catecholamine levels.

Surgery is the treatment of choice; radiotherapy is questionable. Expectation approach and embolization with routine assessments are treatment options for those who do not fit the surgery profile^4^, as was our patient.

## CONCLUSION

Surgery is the major therapeutic approach. Radiotherapy, controversial, did not prove to help in these tumors. To wait and use embolization also seems to be an option, and this was the approach preferred by our patient because of his cardiac condition, and it was an excellent outcome.
